# Chicken or the egg: ST elevation in lead aVR or SYNTA X score

**DOI:** 10.5830/CVJA-2016-062

**Published:** 2017

**Authors:** Levent Cerit

**Affiliations:** Near East University Hospital, Nicosia, Cyprus

**Keywords:** SYNTAX score,, electrocardiography,, lead aVR

## Abstract

**Background:**

ST-segment elevation in lead aVR (STEaVR) anticipates left main and/or three-vessel disease (LM/3VD) in patients with acute coronary syndromes. STEaVR is generally reciprocal to and accompanied by ST-segment depression (STD) in the precordial leads. SYNTAX score (SS) is an angiographic scoring system and is widely used to evaluate the severity and complexity of coronary artery disease. The purpose of our study was to assess the relationship between STEaVR and SS.

**Methods:**

We performed a retrospective analysis of 117 patients with non-ST-segment elevation acute coronary syndrome (NSTEACS). Electrocardiograms at presentation were reviewed, especially for ST-segment elevation of ≥ 0.05 mV in lead aVR and STD of ≥ 0.05 mV in more than two contiguous leads. All lesions causing ≥ 50% stenosis in a coronary artery with a diameter of ≥ 1.5 mm were included in the SS calculation. SS was divided into two groups: ≥ 23: high, < 23: low.

**Results:**

Among the 117 patients, 80 (68.4%) had STEaVR and 37 (31.6%) did not. Patients with STEaVR had a higher SS and higher rate of LM/3VD (85 vs 67.6%, p < 0.001; 86.2 vs 72.9%, p = 0.03, respectively) than those without STEaVR. On multivariate analysis, STEaVR [odds ratio (OR) 1.85; 95% confidence interval (CI): 1.20–3.97, p = 0.03] and STD in leads V_1_–V_4_ (OR 2.14; 95% CI: 1.46–4.23, p = 0.002) were independent predictors of a high SS.

**Conclusion:**

This study demonstrated that STEaVR was an independent predictor of a high SS.

## Background

Previous studies have shown the independent predictive value of ST-segment elevation in lead aVR (STEaVR) for left main and/or three-vessel disease (LM/3VD) in non-ST-segment elevation acute coronary syndrome (NSTEACS).^1^,^2^ STEaVR is generally reciprocal to and accompanied by ST-segment depression (STD) in the precordial leads. Patients with acute coronary syndrome resulting from LM/3VD are at high risk of short- and long-term adverse cardiovascular events.^3^-^5^ Previous studies have assessed the independent predictive value of STEaVR for LM/3VD in NSTEACS and have reported conflicting results.^1^,^2^

SYNTAX score (SS) is a recently developed angiographic grading tool to evaluate the complexity of coronary artery disease. It is widely used for determining the optimal revascularisation strategy. It is also a powerful stratification mechanism, allowing uniform, standardised assessment of the extent and severity of coronary artery disease.6 The purpose of this study was to assess the relationship between STEaVR and SS in patients with NSTEACS.

## Methods

A retrospective analysis was performed on all patients who had undergone coronary angiography and coronary artery bypass grafting (CABG) between January 2013 and January 2016 at the Near East University Hospital. Myocardial infarction (MI) was diagnosed according to the criteria of the European Society of Cardiology and American College of Cardiology.^7^

Inclusion criteria for the study were troponin level greater than the 99th percentile reference value before cardiac catheterisation, chest pain or ischaemic changes on the electrocardiogram (ECG), including horizontal or down-sloping STD (≥ 0.05 mV), and absence of ST-segment elevation on the ECG. Exclusion criteria were previous CABG, bundle branch block or ventricular pace rhythm, severe aortic stenosis, hypertrophic cardiomyopathy, cardiac arrest on presentation, ventricular tachycardia, supraventricular tachycardia with heart rate greater than 160 beats per min, implantable cardioverter defibrillator shock, subsequent documented diagnosis of Takotsubo cardiomyopathy, myocarditis or pulmonary embolism.

The study was approved by the local ethics committee. Patients’ demographic data and risk factors, including current smoking, diabetes mellitus (DM), hypertension (HT), hyperlipidaemia, previous MI, and previous percutaneous coronary intervention were obtained from medical records.

Cardiac troponin T (cTnT) levels were measured using the electrochemiluminescence immunoassay method (Roche Cobas E601). The upper limit of normal for cTnT was 0.014 ng/ml, which represented the 99th percentile reference value. cTnT was measured serially at intervals of approximately four hours, both before and after catheterisation as clinically indicated, with the highest level noted as the peak cTnT.

Two independent, blinded physicians reviewed ECGs obtained at presentation. In the event of an interpretative discrepancy, a consensus between reviewers was reached through discussion.

ST-segment shifts were measured at the J point for ST-segment elevation and depression. STD of ≥ 0.05 mV in more than two contiguous leads was recorded. A cut-off value of ≥ 0.05 mV for STD was chosen, in line with the current universal definition of MI.8 The location of STD was recorded as the anterior (V_1_–V_4_), lateral (I, aVL, V_5_ and V_6_) and inferior (II, III and aVF) region. STEaVR of ≥ 0.05 mV was recorded. Transthoracic echocardiography was performed in a standard manner during hospitalisation, and left ventricular ejection fraction (LVEF) was calculated using the biplane Simpson’s method.

All patients underwent cardiac catheterisation within five days of presentation with NSTEACS. All patients underwent CABG within two weeks of presentation with NSTEACS. An independent cardiologist blinded to the clinical data reviewed all coronary angiography results for the purposes of comparative assessment with the primary treating cardiologist.

## Coronary angiography and SYNTAX score analysis

Coronary angiography was performed by the Judkins technique. All lesions causing ≥ 50% stenosis in a coronary artery with a diameter of ≥ 1.5 mm were included in the SS calculation. For calculation, the website software (http://www.SYNTAXcore.com) was used.

The score was calculated for each patient with regard to the following parameters: coronary dominance, number of lesions, segments included per lesion, the presence of total occlusion, bifurcation, trifurcation, aorto-osteal lesion, severe tortuosity, calcification, thrombus, diffuse/small-vessel disease, and lesion length > 20 mm. SS was calculated separately by two interventional cardiologists blinded to the study protocol and patient characteristics. In the case of a contradiction between two results, the opinion of a senior interventional cardiologist was applied and a common consensus was obtained. SS was divided into two groups: ≥ 23: high, < 23: low

## Statistical analysis

Statistical analysis was performed using the SPSS (version 20.0, SPSS Inc, Chicago, Illinois) software package. Continuous variables are expressed as mean ± standard deviation (mean ± SD) and categorical variables are expressed as percentage (%). The Kolmogorov–Smirnov test was used to evaluate the distribution of variables. Student’s t-test was used to evaluate continuous variables showing a normal distribution, and the Mann–Whitney U-test was used to evaluate variables that did not show a normal distribution. A p-value < 0.05 was considered statistically significant.

To identify predictors of increased SS, the following variables were initially assessed in a univariate model: age, hypertension, diabetes, STD in anterior, lateral and inferior leads, and STEaVR. Significant variables in univariate analysis were then entered into a multivariate logistic regression analysis using backward stepwise selection.

## Results

A total of 117 patients who underwent coronary angiography within five days and CABG within two weeks of presentation with the diagnosis of NSTEACS were included in the analysis. Among the 117 patients, 80 (68.4%) had a STEaVR of ≥ 0.05 mV.

The patients’ characteristics are summarised and presented in Table 1. Patients with STEaVR were older, with a higher peak cTnT value (Table 1). With regard to ECG findings, patients with a STEaVR were more likely to have concomitant STD. Among 80 patients with STEaVR, 68 presented with concomitant STD, comprising anterior (56 patients), lateral (62 patients) and inferior (45 patients) STD (Table 1).

**Table 1:** General characteristics of the patients

	ST elevation in lead aVR		
Patient characteristics	+ (n = 80) (68.4%)	– (n = 37) (31.6%)	p-value
Age, years	63.3 ± 7.4	59.4 ± 8.1	0.027
Male gender, n (%)	27 (73.0)	62 (77.5)	0.485
Hypertension, n (%)	24 (64.8)	51(63.7)	0.352
Diabetes mellitus, n (%)	38 (47.5)	13 (35.1)	< 0.001
Current smoking, n (%)	33 (41.2)	14 (37.9)	0.754
SYNTAX score	27.4 ± 4.9	23.1 ± 5.4	0.002
High SYNTAX score ratio, n (%)	68 (86)	25 (67.6)	< 0.001
Inferior ST-segment depression, n (%)	45 (56.2)	12 (32.4)	< 0.001
Lateral ST-segment depression, n (%)	62 (77)	14 (37.8)	< 0.001
Anterior ST-segment depression, n (%)	56 (70)	14 (37.8)	< 0.001
Left ventricular ejection fraction (%)	58.5 ± 4.2	61.7 ± 5.6	0.652
Left main/three-vessel disease, n (%)	69 (86.2)	27 (72.9)	0.03
Peak troponin T (ng/ml)	1.8 ± 0.5	0.36 ± 0.12	0.002

Patients with STaVR had a significantly higher rate of LM/3VD and higher SS than those without STEaVR (86.2 vs 72.9%, p = 0.03; 85 vs 67.6%, p < 0.001, respectively) (Table 1). The results of univariate analysis are presented in Table 2. On univariate analysis, age, HT, DM, ST-segment elevation in lead aVR and STD in the anterior, lateral and inferior leads were associated with a high SS (Table 2). On multivariate analysis STEaVR and STD in the anterior leads were independent predictors for a high SS (OR 2.12; 95% CI: 1.34–4.13, p < 0.001; OR 1.64; 95% CI: 1.24–2.86, p = 0.02, respectively) (Table 3).

**Table 2:** Univariate analysis of predictors for a high SYNTA X score

Predictor variables	OR (95% CI)	p-value
Age	2.723 (1.534–4.842)	< 0.001
Diabetes mellitus	1.246 (0.827–1.543)	0.54
Hypertension	1.14 (0.784–1.457)	0.37
Inferior ST-segment depression	1.924 (1.465–3.147)	< 0.001
Lateral ST-segment depression	2.416 (1.354–4.249)	< 0.001
Anterior ST-segment depression	2.160 (1.527–3.895)	< 0.001
ST-elevation in lead aVR	3.012 (1.974–4.243)	< 0.001

**Table 3:** Multivariate analysis of predictors for high SYNTA X score

Predictor variables	OR (95% CI)	p-value
Age	1.23 (0.652–1.524)	0.42
Inferior ST-segment depression	1.324 (0.465–2.862)	0.39
Lateral ST-segment depression	2.351 (1.524–4.243)	< 0.001
Anterior ST-segment depression	1.214 (0.527–1.253)	0.48
ST elevation in lead aVR	2.827 (1.873–4.368)	< 0.001

## Discussion

Our study showed that STEaVR and STD in the anterior leads were independently associated with a high SS and higher rates of LM/3VD in patients with NSTEACS. To our knowledge, this is the first study to evaluate STEaVR in patients with NSTEACS who underwent coronary angiography followed by CABG surgery.

Previous studies have reported the independent predictive value of STEaVR for LM/3VD in NSTEACS. Barbares et al.^9^ reported that patients with STEaVR had a higher prevalence of LM/3VD and increased risk of in-hospital death. Kosuge et al.^1^ showed that STEaVR (≥ 0.05mV) was independently associated with LM/3VD, and STEaVR and increased cTnT level were independent predictors of death or MI only in patients with NSTEMI. Rostoff et al.^10^ evaluated the prognostic role of STEaVR in 134 patients with NSTEACS and reported that left main coronary artery disease was independently associated with STEaVR.

Atie et al.^8^ evaluated ECG changes in patients with left main disease and showed that the most frequently observed ECG finding was STD in leads V_3_, V_4_ and V_5_. In addition to the predictive value for LM/3VD, STD carries a significant prognostic value in patients with NSTEMI.^11^,^12^ Furthermore, STD in leads V_4_–V_6_ has been reported to be an independent predictor for short-term mortality in patients with inferior ST-elevation myocardial infarction (STEMI).^13^ In the present study, STD in the anterior leads was another independent predictor for a high SS.

Janata et al.^14^ has shown the prognostic value of STEaVR in patients with acute pulmonary embolism. In our study pulmonary embolism was excluded by echocardiographic and biochemical findings. One of the most common causes of STEaVR is left ventricular hypertrophy (LVH), which may represent repolarisation abnormalities.^15^ In our study LVH was excluded by echocardiographic evaluation.

Although major STEaVR (> 0.1 mV) remained an independent predictor of LM/3VD, minor (0.05–0.1 mV) and major STEaVR were not independent predictors of in-hospital or six-month death, after adjusting for other validated prognosticators in the GRACE risk model.^2^ Taglieri et al.16 investigated the prognostic significance of STEaVR in patients with NSTEMI. They reported that STD plus STEaVR were associated with highrisk coronary lesions and predicted in-hospital and one-year cardiovascular death. Several studies have reported a close relationship between STEaVR and in-hospital or one-year cardiovascular death.^1^,^9^,^16^ In this study, we could not evaluate the relationship with mortality due to lack of data.

SS, which is used in the evaluation of angiographic severity and extent of coronary lesions, has been shown to predict mortality in addition to its role in the decision-making process of interventional procedure.^6^,^17^ SS predicted short- and longterm adverse events following revascularisation in a study by Valgimigli and co-workers.^18^ In our study, STEaVR was an independent predictor of increased SS. It is well known that SS predicts mortality after a revascularisation procedure.

Nabati et al.^19^ reported that STEaVR was independently associated with severity of coronary artery atherosclerosis and decreased LVEF in patients with NSTEACS. Although we found a significant relationship between STEaVR and severity and extent of coronary artery disease, there was no difference regarding LVEF. Additionally, they have shown that this ECG pattern had been associated with markers of myocardial necrosis and high-risk coronary lesions, including multi- or three-vessel coronary artery disease.^19^ Similarly, in our study, there was a significant difference with regard to peak cTnT value in patients with STEaVR.

Several studies have shown a different ratio of STEaVR in patients with NSTEACS; Barrabés et al.^9^ reported 32.2%, Kosuge et al.^5^ reported 27.4%, Taglieri et al.^16^ reported 15.7%, Misumida et al.^20^ reported 26%, and Nabati et al.^21^ reported 40.3%. In our study, the ratio of STEaVAR was 68.4%. Misumida et al.^20^ reported that patients with STEaVR had a significantly higher rate of LM/3VD than those without STEaVR (39 vs 18%, respectively, p < 0.001). Nabati et al.^21^ reported that patients with STEaVR had a significantly higher rate of three- or multi-vessel disease than those without STEaVR (53.8 vs 31.2%, respectively, p = 0.01).

In our study, patients with STEaVR had a significantly higher rate of LM/3VD than those without STEaVR (86.2 vs 72.9%, respectively, p = 0.03). High rates of STEaVR and LM/3VD in our study are thought to have resulted from the inclusion of CABG patients into the study.

There are several limitations in this study. First, our study was a retrospective, observational study. Second, the sample size was small. Third, we did not exclude patients with posterior infarction presenting with STD in V_1_–V_4_, which is equivalent of STEMI. Therefore, our study group may have included patients with posterior STEMI. Fourth, we could not access death records in our country, therefore we could not evaluate mortality rates in this study.

## Conclusion

This study demonstrates that STEaVR and STD in the anterior leads were independent predictors of a higher SS and higher rate of LM/3VD in patients with NSTEACS.
